# Assessment of ENI Gemeni microprocessor-controlled centrifugal analyser

**DOI:** 10.1155/S1463924679000572

**Published:** 1979

**Authors:** N. Potezny, R. G. White, T. D. Geary

**Affiliations:** Institute of Medical and Veterinary Science Box 14 Rundle Street PO Adelaide South Australia 5000 Australia


for each step of the analysis cycle. At the end of each step the external clock is reset as is the 8 bit control pattern for the next function. The final step is to activate the AA analysis cycle and to wait for the furnace to cool before injection of the next sample. We have found that programming a waiting time longer than the cooling time extends the life of the graphite cuvettes. The program for operation in the pulsed mode is given in Table 1.

The second injection is a critical part of the analysis cycle for non-metals such as selenium and arsenic. The addition of a co-analyte such as $Ni^{2+}$ has been shown to increase the sensitivity for these elements and reduces loss due to the volatility of these elements [1, 5]. The syringe pump used for the second injection delivers 11.0 ± 0.1 μl/sec of co-analyte. The same syringe pump is used for the total consumption analysis mode. In the total consumption mode, however, the analyte is pumped out of the holding tube into the sampling valve and the dispensing of the sample then proceeds in the same fashion as in the pulsed mode of operation. The software modification for total consumption analysis involves only increasing the second injection time to 4-8 seconds and performing the second injection prior to the sample injection.

The graphite cuvette volume in the currently employed AA system is approximately 50 μl and the standard pulsed mode experiment employs 37 μl ± 1.7 μl analyte from the sampling loop and 11.0 μl + 0.1 μl of co-analyte from the syringe pump. The total consumption mode pumps 100 μl of eluent into the sampling loop and 37 μl are then dispensed into the graphite furnace. This 'overrun' ensures the complete filling of the sample loop and causes no problem in subsequent interpretation of data.

### Conclusion

The use of an inexpensive microprocessor system adds a great deal of versatility to a previously "hard wired" LCAA sampling system [1]. Both pulsed and total consumption analyses are possible with only minor plumbing and software changes. The use of other microprocessor systems would require only a change in machine language. The system has the advantage of being inexpensive. The interface sampling system can be assembled for a component cost of approximately $500 (depending on the availability of surplus equipment).

With the many advantages of performing LCAA analysis on trace level metal-containing compounds, hopefully this technique will find widespread use with investigators in the clinical, environmental, and inorganic biochemistry fields.

ACKNOWLEDGEMENT

We wish to thank April Robertson for technical assistance and the Robert A. Welch Foundation (Grant No. A-694) for financial support.

REFERENCES

[1] Vickrey, T.M. , Buren, M.S., Howell, H.E., *Analytical Letters*, 1978, *A11*, 1075-1095.
[2] Brinckman, F.E., Blair, W.R., Jewett, D.L., and Iverson, W.P., *J. Chrom. Sci.*, 1977, 15, 495-503, and references therein.
[3] Vickrey, T.M., Howell, H.E., Paradise, M.T., *Analytical Chemistry*, submitted.
[4] Slavin, W., Schmidt, G.J., Abstract No. 126, "Pittsburgh Conference on Analytical Chemistry and Applied Spectroscopy", Cleveland, Ohio, March 1979.
[5] Pierce, F.G., and Brown, H.R., *Analytical Chemistry*, 1977, *49*, 1417-1421.

### Assessment of ENI Gemeni microprocessor-controlled centrifugal analyser

**N. Potezny, R.G. White, and T.D. Geary**

*Institute of Medical and Veterinary Science, Box 14, Rundle Street PO, Adelaide, South Australia, 5000.*

The ENI Gemeni was assessed in this institute for its suitability as a general laboratory instrument using a procedure which has been developed during the last two years for the Committee for Evaluation of Kits and Instrumentation of the Australian Association of Clinical Biochemists.

### Materials and Methods

The Gemeni is a miniature centrifugal analyser consisting of an analyser module, microprocessor and work station.

The methods recommended by the manufacturer for use on the Gemeni were run in parallel with routine methods used in this laboratory. These routine methods were:

(1) Glucose – Glucose Analyser, Yellow Springs Inst. Co. (YSI).
(2) Cholesterol – Abbott Agent Enzymatic Reagent, Abbott ABA 100.
(3) Calcium – Cresolphthalein complexone, SMAC, Technicon Equipment Pty. Ltd.
(4) Alkaline
(5) Phosphatase – p-nitrophenol, Beckman TR.

These methods have known accuracies and precisions. The Center of Disease Control, Atlanta, USA, (CDC) hexokinase reference method was used to check the YSI glucose analyser. The cholesterol method has been standardised against the WHO Lipid Standardisation Laboratory at CDC. The calcium method has been compared with the Cali, Young and Bowers [1] method using materials from the Massachusetts Society of Pathology with definitive values assigned by an isotope dilution technique.

The method of assessment was based upon the work of Tonks [2], Barnett and Youden [3], Logan [4], Broughton et al [5] and modified in the light of our own experience.

### Precision

(a) Intrabatch imprecision was checked by the analyses of replicates in the same batch and duplicates on the patient comparisons.

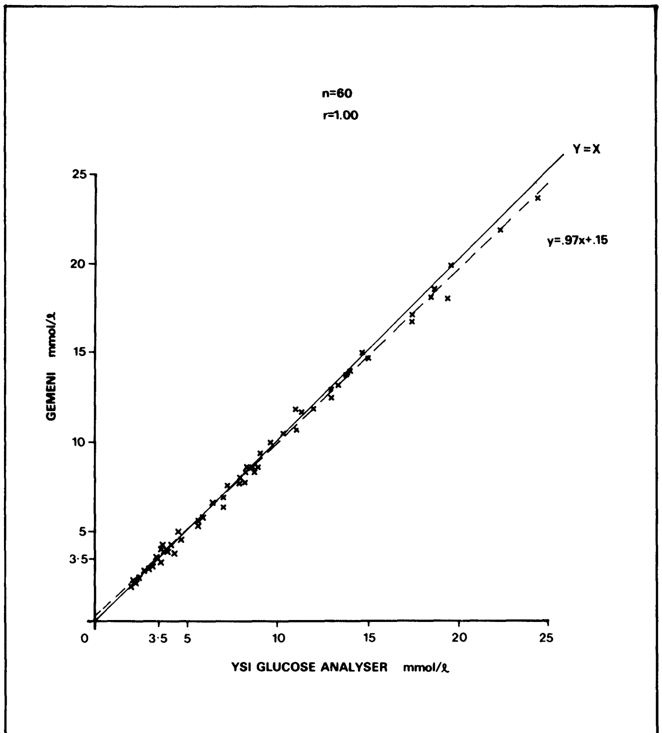

*Figure 1. Glucose*

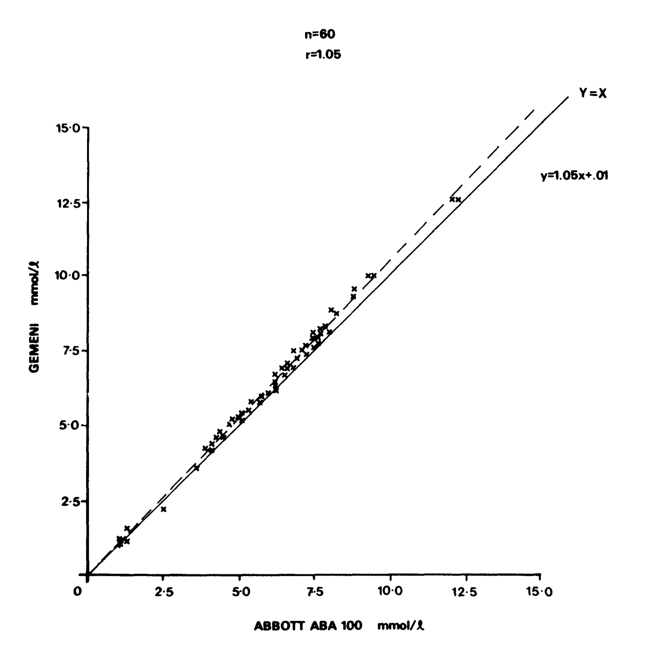

*Figure 2. Cholesterol*

(b) Interbatch imprecision was assessed by analysing control materials at three levels, twice a day for a period of ten days. The materials were selected to coincide with decision levels, and to cover the linear range of the analyte to be determined.

### Recovery

Constituents were added to pooled sera so that the final concentrations represented typical ranges of normal and abnormal values encountered in patient sera.

### Accuracy

Bias was checked by comparing patient samples against a reference method or one of known bias. These were used in conjunction with quality control material with consensus values from the Comprehensive Chemistry Quality Assurance Programme, College of American Pathologists, or materials with assigned values from CDC. As a further guide to accuracy a number of commercial control materials were assayed. The results tabulated for quality control material are the means of triplicate analysis.

**Table 1**
**Intrabatch Imprecision**

| | Glucose mmol/l | Cholesterol mmol/l | Calcium mmol/l | Alkaline Phosphatase U/l |
|---|---|---|---|---|
| Mean | 9.36 | 4.42 | 2.47 | 102.9 |
| SD | 0.11 | 0.06 | 0.05 | 2.63 |
| CV% | 1.18 | 1.43 | 1.90 | 2.56 |

**Table 2**
**Interbatch Imprecision**

| | Mean | SD | 2 CV% | ALE |
|---|---|---|---|---|
| Glucose mmol/l | | | | |
| Low | 2.16 | 0.12 | 11.12 | 10.0 |
| Medium | 6.11 | 0.24 | 7.86 | 10.0 |
| High | 18.67 | 0.39 | 4.18 | 10.0 |
| Cholesterol mmol/l | | | | |
| Low | 2.28 | 0.06 | 5.26 | 10.0 |
| Medium | 6.10 | 0.19 | 6.22 | 10.0 |
| High | 12.75 | 0.39 | 6.12 | 10.0 |
| Calcium mmol/l | | | | |
| Low | 1.73 | 0.07 | 8.10 | 6.0 |
| Medium | 2.66 | 0.08 | 6.10 | 6.0 |
| High | 3.70 | 0.08 | 4.32 | 6.0 |
| Alkaline Phosphatase U/1 | | | | |
| Low | 29.3 | 1.87 | 12.76 | 20.0 |
| Medium | 73.3 | 4.08 | 11.14 | 20.0 |
| High | 261.2 | 9.02 | 6.90 | 20.0 |

### Linearity

Linearity was checked for the four parameters assayed. For glucose, cholesterol and calcium this was achieved by assaying aqueous standards, while for alkaline phosphatase a high patient serum was diluted in pooled sera with low values.

### Literature score

The literature score was obtained by the method of Krynski and Logan [6] using the recommendations of the American Association of Clinical Chemists Committee on Standards [7].

### Miscellaneous tests

The precision and accuracy of the reagent dispenser was checked.

The precision and accuracy of delivering a pre-set volume of reagent was determined at two volume settings. This was achieved by dispensing set volumes of distilled water into a weighing tray and weighing the amount of water dispensed.

Several days were allowed in which to become familiar with the instrument prior to the commencement of the evaluation. The familiarisation protocol consisted of the following points:

(a) Instrument installed by the distributor.
(b) Instrument operated by laboratory staff under the distributor's supervision.
(c) A verification procedure undertaken in the presence of the distributor to check that the instrument was performing to manufacturer's specifications.
(d) Final acceptance by both the distributor and evaluators.

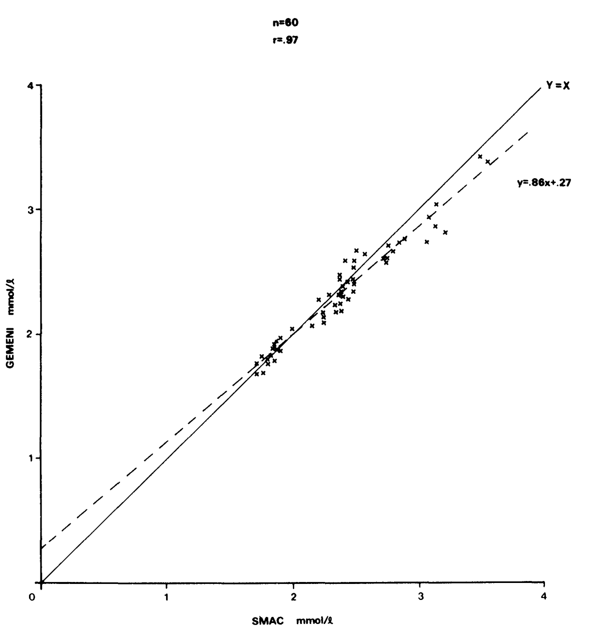

*Figure 3. Calcium*

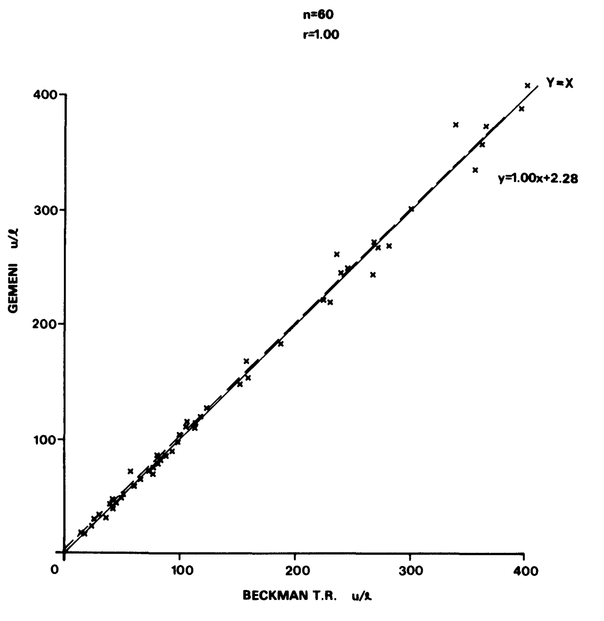

*Figure 4. Alkaline phosphatase.*

### Results

### Precision

(a) Intrabatch imprecision for the analytes tested are given in Table 1.

(b) Interbatch imprecision results are given in Table 2.

From the results in Table 1 and 2, it can be seen that alkaline phosphatase and cholesterol have acceptable degrees of imprecision over the range of values assayed.

Imprecision for glucose was unacceptable by Tonk's criterion 2 CV% < ALE (allowable limits of error) at the low level. However, this level of imprecision at such a low glucose concentration is clinically acceptable. The calcium methodology had unacceptable imprecision at low and medium levels.

### Recovery

Table 3 shows the recovery data for glucose determination. Recovery data were made for glucose only for two reasons. The report is primarily an assessment of the instrument, and not the kits, and the instrument had to be returned to the distributor before a 'complete evaluation' could be completed. The recoveries were acceptable by Logan's criteria, i.e. acceptable recoveries average between 90% and 110% with no single recovery less than 85% or greater than 115%.

### Accuracy

(a) *Patient comparison*

The computer plot of the patient comparison data is shown in Figures 1, 2, 3 and 4. Applying the "t" test, all the Gemeni results except alkaline phosphatase are significantly different from the comparative methods. Results of the "t" test were as follows: glucose 3.30, cholesterol 11.00, calcium 3.68 and alkaline phosphatase 1.5. This must be viewed in its proper context; a result may be statistically significant but clinically acceptable. If the "t" test is significant then further criteria are applied, that is a kit is unacceptable if:

(1) The bias exceeds the allowable limits of error for precision.

(2) The number of false clinical decisions exceeds 5%.

**Table 3**
**Glucose Recovery**

| Sample | Mean Value mmol/l | Recovery % |
|---|---|---|
| Pooled serum | 6.6 | – |
| P+3.0 | 9.4 | 93 |
| P+6.0 | 12.4 | 97 |
| P+12.0 | 18.5 | 99 |

**Table 4**
**Glucose Accuracy**

| Control | Value mmol/l | Observed value |
|---|---|---|
| CDC 1 | 4.3 | 4.3 |
| CDC 2 | 7.3 | 7.3 |
| CAP C1 | 5.6 | 5.5 |
| CAP C2 | 5.8 | 5.8 |
| CAP C3 | 5.7 | 5.6 |
| Monitrol 1 | 4.9 | 4.6 |
| Monitrol 11 | 11.8 | 12.0 |
| QPAK 1 | 5.2 | 5.3 |
| QPAK 11 | 11.3 | 11.3 |
| Versatol A Alternate | 17.2 | 17.1 |

**Table 5**
**Cholesterol Accuracy**

| Control | Value mmol/l | Observed value mmol/l |
|---|---|---|
| Q Pak 1 | 2.9 | 2.75 |
| Q Pak 11 | 2.9 | 2.75 |
| Versatol A Alternate | 2.85 | 2.35 |
| Monitrol 1 | 4.12 | 3.52 |
| Serachol | 10.1 | 10.0 |
| Lipidtrol | 8.1 | 8.4 |
| Calbiochem lipid control A | 11.3 | 11.1 |
| Calbiochem lipid control B | 3.1 | 3.1 |

Table 6
Alkaline Phosphatase Accuracy

| | Value U/l | Observed value U/l |
|---|---|---|
| Q Pak 1 | 109 | 98 |
| Q Pak 11 | 343 | 340 |
| Q Pak multi-enzyme control C | 419 | 347 |
| Monitrol 1 | 40 | 54 |
| Monitrol 11 | 220 | 210 |
| Validate | 195 | 215 |

Table 7
Calcium Accuracy

| Control | Value mmol/l | Observed value |
|---|---|---|
| CAP C1 | 2.45 | 2.40 |
| CAP C2 | 2.58 | 2.50 |
| CAP C3 | 2.45 | 2.45 |
| Validate A | 3.43 | 3.38 |
| Validate | 2.35 | 2.30 |
| Monitrol 1 | 2.45 | 2.43 |
| Monitrol 11 | 2.18 | 2.25 |
| Q Pak 1 | 2.38 | 2.38 |
| Q Pak 11 | 3.35 | 3.18 |
| Versatol A Alternate | 3.20 | 3.08 |

Based on these criteria of acceptability, the results for glucose, cholesterol and alkaline phosphatase are acceptable, while those obtained for calcium.are unacceptable.

(b) *Comparison of results on commercial control material* Bias less than allowable limits of error for precision from a carefully verified reference serum value which relates to all parameters tested, indicates that the test method gives a satisfactory measurement of the "true value". Results obtained for control sera are shown in Tables 4, 5, 6 and 7. These indicate that accuracy is acceptable for glucose, cholesterol and alkaline phosphatase but unacceptable for the calcium methodology.

**Linearity**

Linearity for the methods tested was as follows:

glucose linear to 20 mmol/l cholesterol linear to 15 mmol/l alkaline phosphatase linear to 400 U/l calcium linear to 3.75 mmol/l

**Literature score**

One mark was assigned for each point covered in the information supplied by the manufacturer. One half mark was awarded for each point that had received only partial attention. In case of doubt the scoring favoured the manufacturer.

The Gemeni's literature scored 13 out of a possible 15 points.

**Miscellaneous tests**

*Precision and accuracy of Clay-Adams Selectapette.*
Results obtained were as follows:

(a) Volume pre-set to deliver 1.0 ml
Mean volume delivered 1.000 ml
CV% 0.3

(b) Volume pre-set to deliver 0.5 ml
Mean volume delivered 0.500 ml
CV% 0.4

These results are within the limits of acceptability for accuracy and precision i.e:

(a) accuracy 1% stated volume
(b) precision CV less than 1%

### Discussion

Precision for cholesterol and alkaline phosphatase methodologies was acceptable over the range of values assayed. Glucose precision was acceptable at medium and high concentrations; at low concentrations precision was unacceptable by Tonk's criterion. However, at such low glucose concentration the precision is clinically acceptable. The calcium method proved unacceptable by Tonk's criterion at low and medium levels.

The accuracy for glucose, cholesterol and alkaline phosphatase were acceptable. The results obtained for the calcium kit were unacceptable.

A feature of the instrument which will aid quality control programmes is the print out of a 'quality control factor' and a reagent drift at the end of each run. A draw back with the instrument is its lack of flexibility. The microprocessor program is selected by coded holes in the test card. This makes it impossible to alter parameters in the program and, therefore, difficult to use kits produced by other manufacturers.

The instrument performed creditably throughout the evaluation and proved to be easy to operate, giving precise and accurate results for cholesterol glucose and alkaline phosphatase. However the quality of results from the calcium kits were unacceptable. When the results for the other analytes are considered this would appear to be a fault of the method rather than the instrument.

### References

[1] Cali, J.P., Young, D.S. and Bowers, G.N. A method for the determination of total calcium in serum. *Clinical Chemistry,* 1973, **19**, 1208.

[2] Tonks, D.B. A quality control program for the quantitative clinical chemistry estimation. *Canad. J. Med. Technol.* 1968, **30**, 38.

[3] Barnett, R.N. and W.J. Youden. A Revised Scheme for the Comparison of Quantitative Methods. *Am. J. Clin. Pathol.* 1970, **54**, 454.

[4] Logan, J.E. Evaluation of Commercial Kits. *C.R.C. Critical Reviews in Clin. Lab. Sciences.* 1972, **3**, 271.

[5] Broughton P.M.G., Gowen Lock A.H., McCormack J.J., Neill D.W. A Reviser Scheme for the Evaluation of Automatic Instruments for use in Clinical Chemistry, *Ann. Clin. Biochem.* 1974, **11**, 207.

[6] Krynski, I.A. and J.E. Logan, Observations on Diagnostic Kits for the Determination of Total Cholesterol, *Clin. Biochem.* 1968, **2**, 105.

[7] American Association of Clinical Chemists Committee on Standard and Controls – A.A.C.C. policy regarding reagent sets and kits, *Clin. Chem.* 1966, **12**, 43.

